# Supplementary material for: Atomic-scale phase separation induced clustering of solute atoms
Source: Nat Commun. 2020 Aug 7;11:3934. doi: 10.1038/s41467-020-17826-w (PMC7415157; doi:10.1038/s41467-020-17826-w)
Supplement: Supplementary file 1 — Supplementary Information [file 41467_2020_17826_MOESM1_ESM.docx]

**Supplementary Information**

*for*

**Atomic-scale phase separation induced clustering of solute atoms**

Lianfeng Zou^1#^, Penghui Cao^2^**^#^**, Yinkai Lei^3#^, Dmitri Zakharov^4^, Xianhu Sun^1^, Stephen D. House^5,6^, Langli Luo^1^, Jonathan Li^1^, Yang Yang^7^, Qiyue Yin^1^, Xiaobo Chen^1^, Chaoran Li^1^, Hailang Qin^1^, Eric A. Stach^8^, Judith C Yang^5,6^, Guofeng Wang^3^, Guangwen Zhou^1*^

^1^Department of Mechanical Engineering & Materials Science and Engineering Program, State University of New York at Binghamton, NY 13902, USA

^2^Department of Mechanical and Aerospace Engineering, University of California, Irvine, Irvine, CA 92697, USA

^3^Department of Mechanical Engineering and Materials Science, University of Pittsburgh, Pittsburgh, PA 15261, USA

^4^Center for Functional Nanomaterials, Brookhaven National Laboratory, Upton, NY 11973

^5^Department of Chemical and Petroleum Engineering, University of Pittsburgh, Pittsburgh, PA 15261, USA

^6^Environmental TEM Catalysis Consortium (ECC), University of Pittsburgh, Pittsburgh, PA 15261

^7^Department of Nuclear Science and Engineering, Massachusetts Institute of Technology, Cambridge, Massachusetts 02139, USA

#### ^8^Department of Materials Science and Engineering, University of Pennsylvania, Philadelphia, PA, 19104, USA

^#^these authors contribute equally to the work

^*^Correspondence to: [gzhou@binghamton.edu](mailto:gzhou@binghamton.edu)


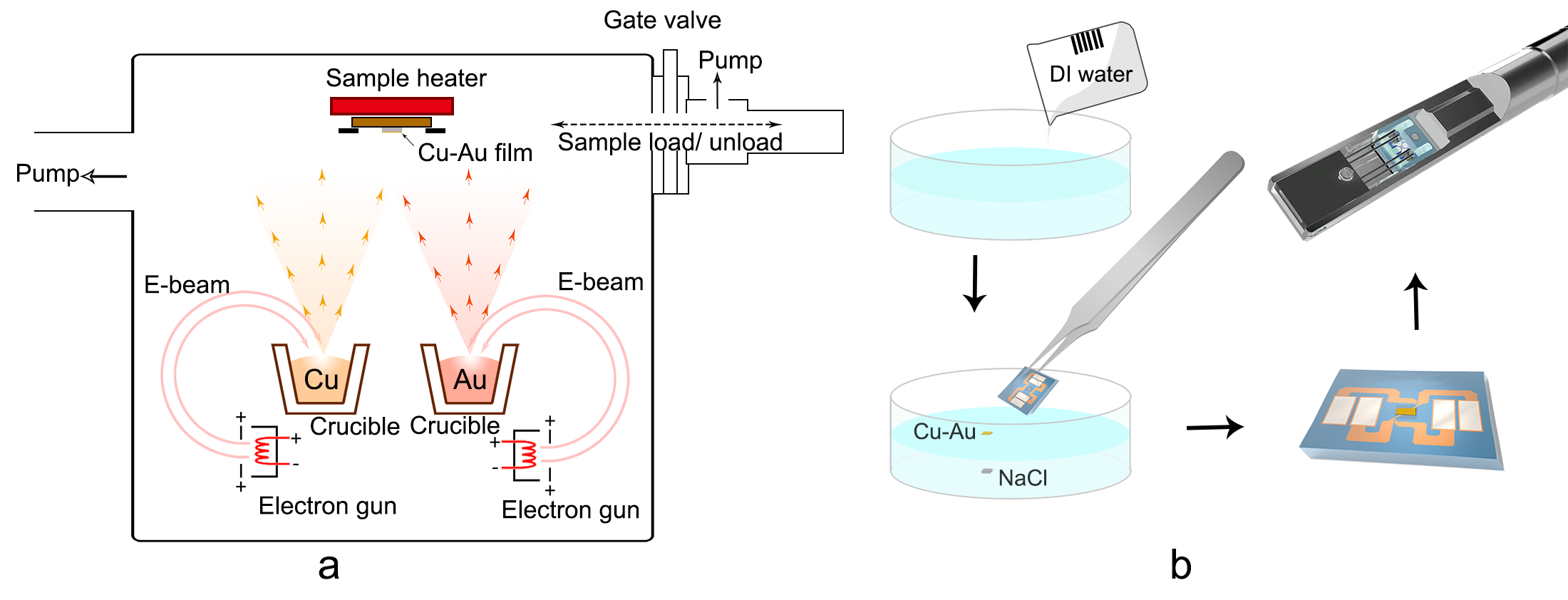


**Supplementary Figure 1:** **Experimental procedure.** **(a)** Schematic of the electron-beam evaporation of Cu-Au thin films on NiAl(100), where the film composition is controlled by manipulating the evaporation rate of Cu and Au in the crucibles. **(b)** The as-prepared Cu-10at.%Au film is then removed from the NaAl substrate by dissolution of the NaCl in deionized water, washed, and mounted on a TEM specimen holder.


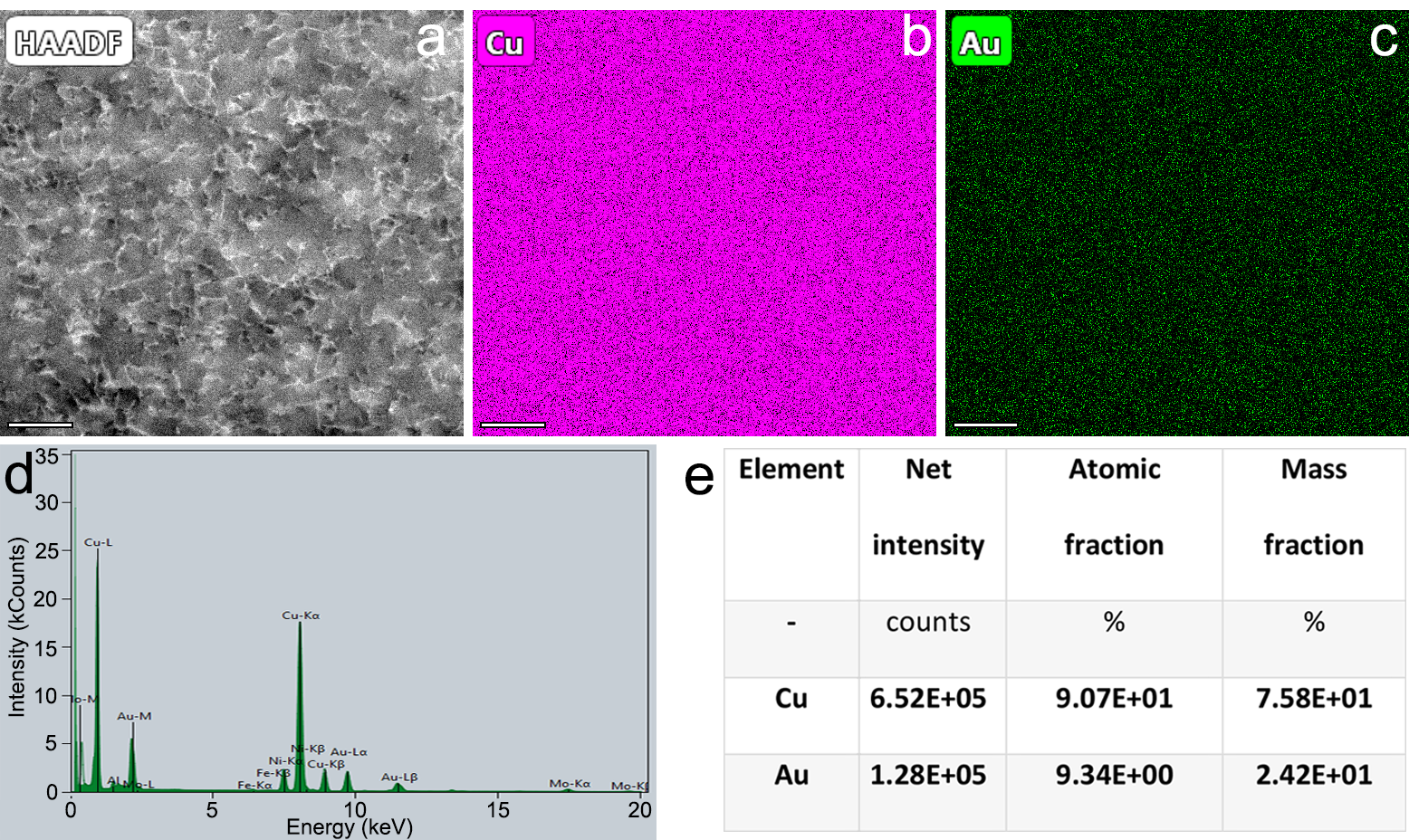


**Supplementary Figure 2: STEM-EDS analysis of the as-prepared Cu-10at.%Au(100) thin films.** **(a)** Low-magnification HAADF image showing the good film continuity over the large area. **(b, c)** STEM-EDS mapping showing the uniform distribution of Cu and Au, respectively. **(d)** Representative EDS spectrum of the as-prepared film. **(e)** Chemical composition by quantification of EDS data, showing an atomic ratio of 90.7%Cu and 9.3%Au, which is very close to the targeted composition of the Cu-10at.%Au film made by e-beam co-evaporation of Cu and Au, where the alloy composition was controlled by manipulating the evaporation rate of Cu and Au. Scale bar, 200 nm **a, b, c**.


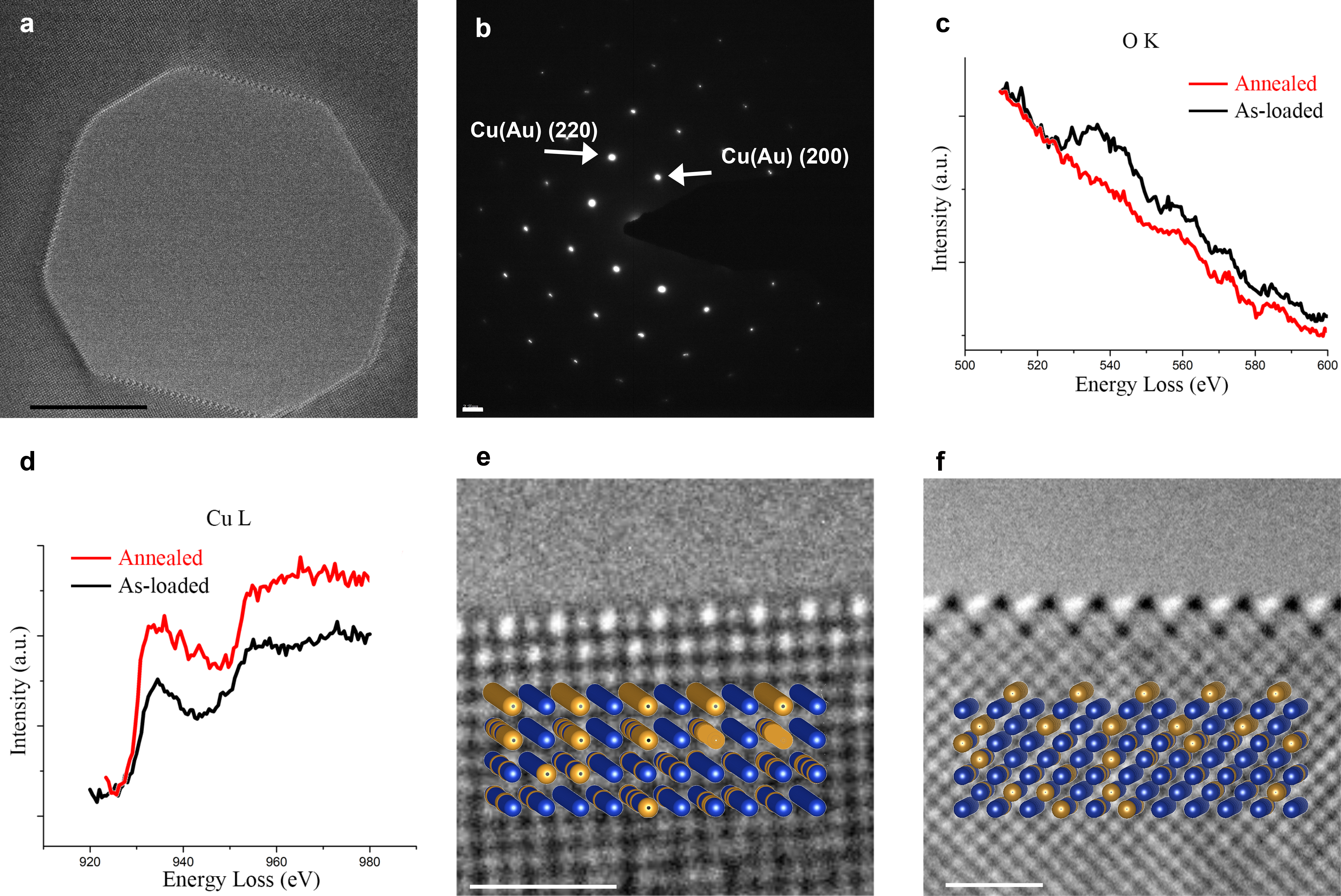


**Supplementary Figure 3:** **TEM characterization of Cu-10at.%A(100) film.** The films were annealed at ~ 350 °C and ~ 0.001 Torr of H_2_ gas flow. **(a)** A representative faceted hole formed in the annealed film, the side facets are typically composed of {100} and {110} surface terminations. **(b)** Electron diffraction pattern along the [001] zone axis, displaying the single crystalline feature of the film, the absence of additional spots confirms the complete removal of native oxide by annealing in H_2_ gas flow. **(c, d)** EELS O-K edge and Cu-L2,3 edges showing the presence of oxygen in the thin film before annealing and the absence of oxygen after annealing. The black and red ones are obtained from the unannealed and annealed sample, respectively. **(e, f)** HRTEM images showing the formation of an ordered Cu_3_Au-like surface alloy along the (100) and (110) side facets as shown in **a**. Scale bar, 10 nm **a,** 2 1/ nm **b,** 1 nm **e, f.**


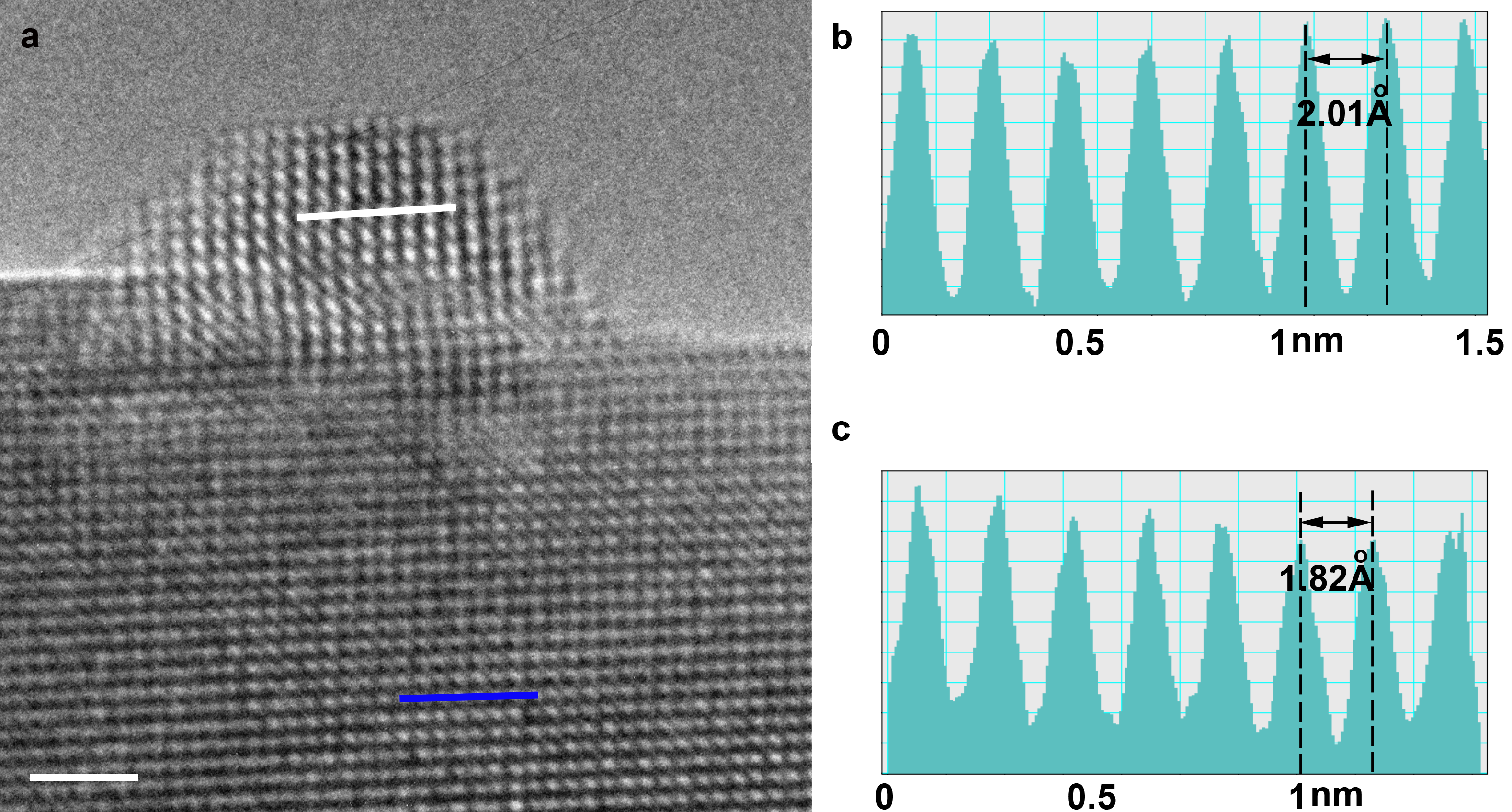


**Supplementary Figure 4:** **Measuring the lattice spacing of Au clusters.** **(a)** HRTEM image of a Au cluster formed by annealing a Cu-10at.%Au(100) film at 600 °C and 1×10^-3^ Torr of H_2_ gas flow. **(b-c)** Intensity profiles along the white and blue lines in **a**, showing that the d(200) spacings for the Au cluster and the Cu-Au substrate are 2.01 Å and 1.82 Å, respectively. Scale bar, 1 nm **a.**


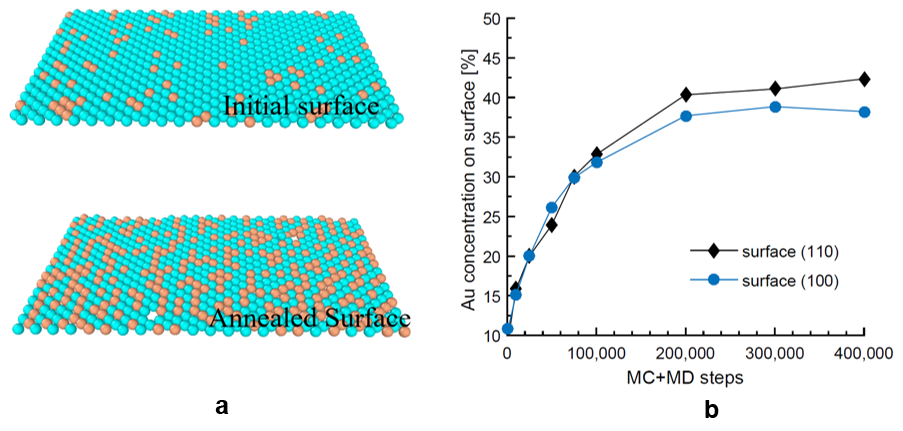


**Supplementary Figure 5:** **Au surface segregation in the Cu-10at.%Au system**. **(a)** The initial and final states of the (100) substrate surface layer from hybrid MC+MD simulations (blue and orange balls represent Cu and Au atoms in the substrate surface, respectively). **(b)** Au concentration in the substrate surface layer for the (100) and (110) surfaces as a function of MC trial steps.


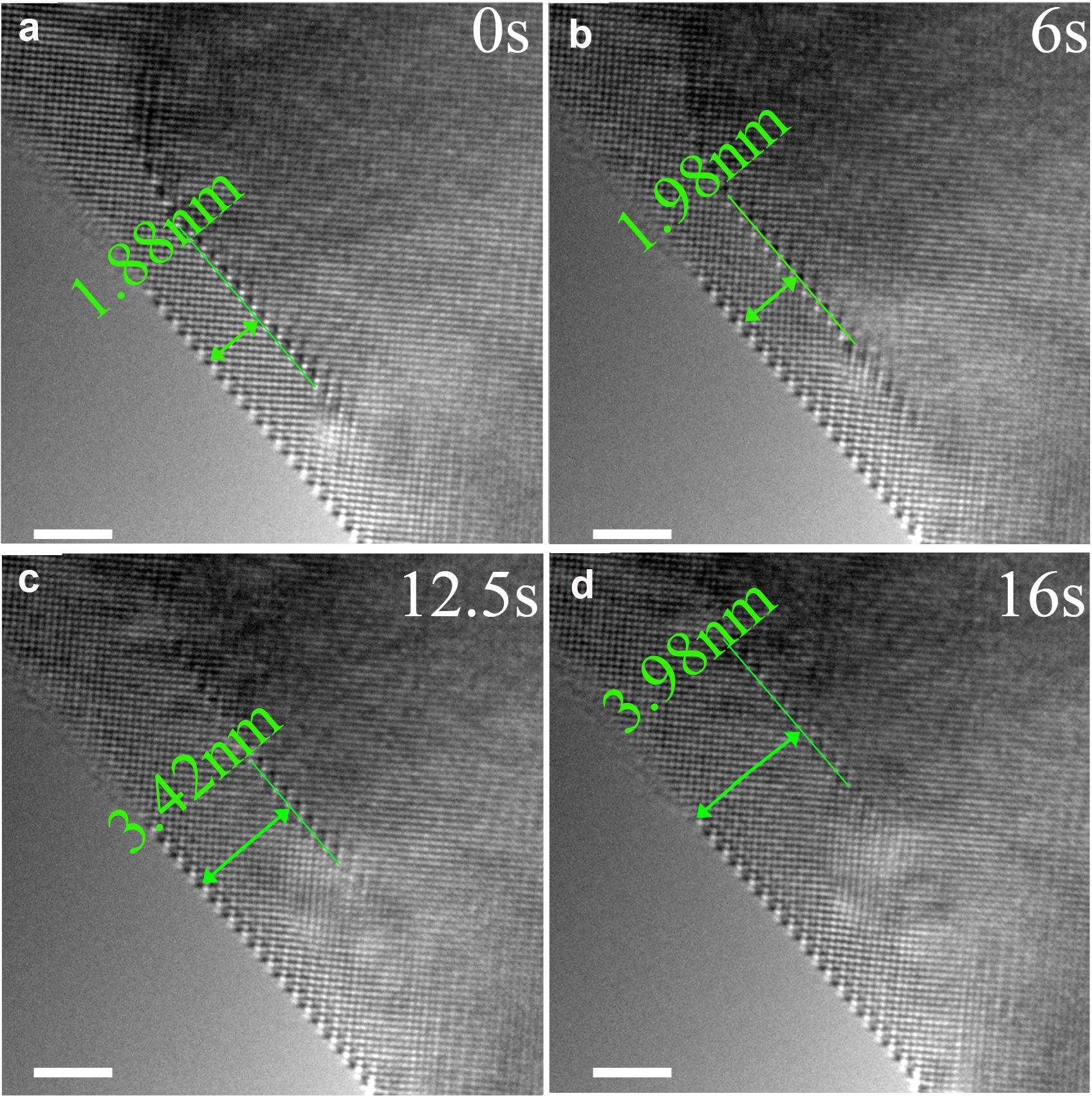


**Supplementary Figure 6: Surface steps act as active sources of Cu and Au adatoms**. **(a-d)** *In situ* TEM observations showing the retraction motion of surface steps during the annealing of a Cu-10at.%Au(100) film at 600 °C and 1×10^-3^ Torr of H_2_ gas flow, which results in a flux of Cu and Au adatoms on the planar surface. Scale bar, 2 nm **a-d.**


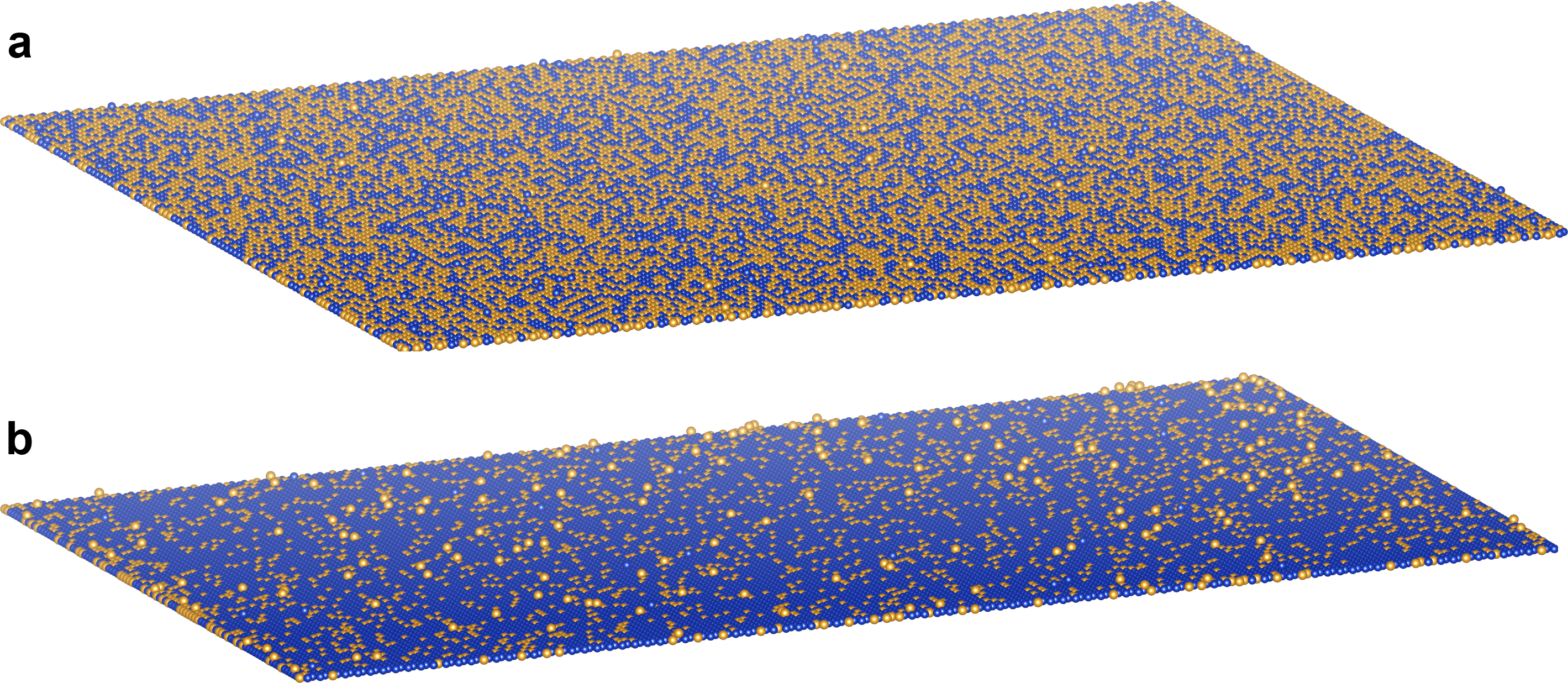


**Supplementary Figure 7: Composition evolution of the fluid phase and the substrate surface**. **(a)** The initial substrate surface with a concentration of 50%Cu-50%Au. **(b)** The substrate surface after 4.9×10^-4^ s, in which the Au concentration in the substrate surface decreases from 50% to 14%. Golden and blue spheres represent Au and Cu atoms, respectively.


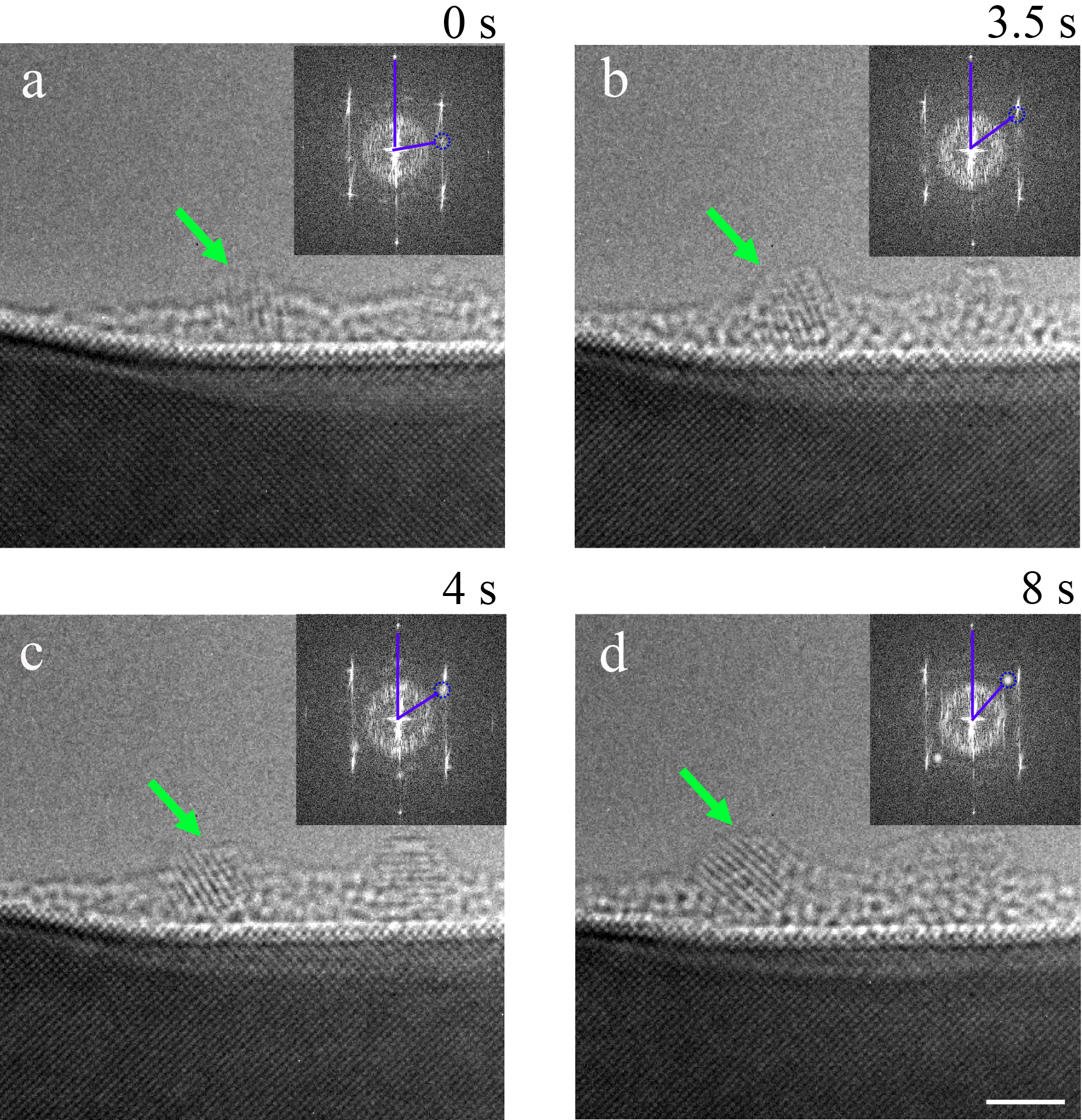


**Supplementary Figure 8:** **Measurement of in-plane rotation of Au clusters**. **(a-d)** In-situ TEM images showing the rotation of an Au cluster pointed by the green arrow. The blue ring circles out one of the diffraction spot pair associated with the Au cluster. The angle between two blue lines represents the orientation between the Au cluster and the Cu(Au) substrate. Scale bar, 4 nm **a-d**.


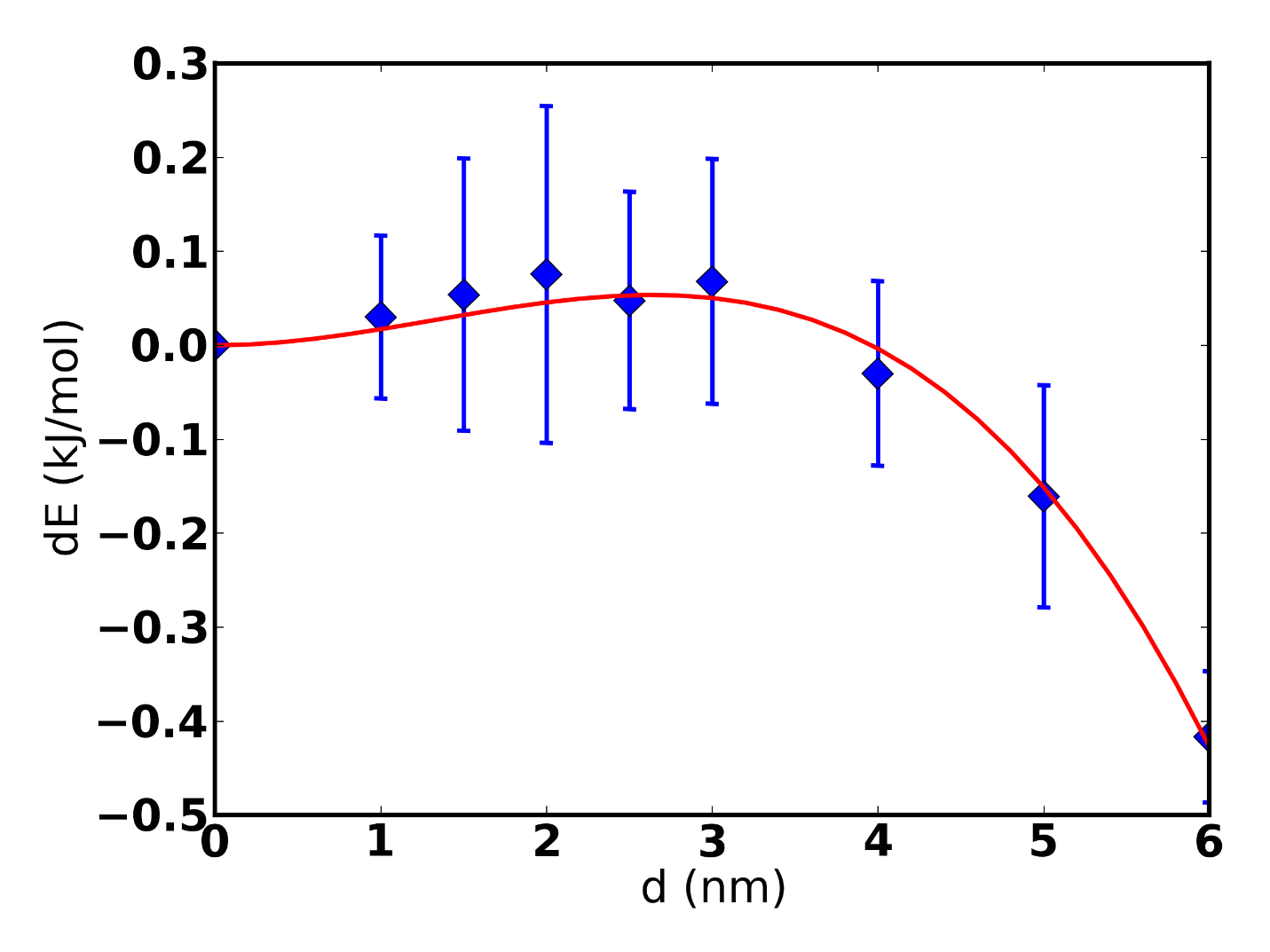


**Supplementary Figure 9: Critical size for the amorphous to crystalline transition in Au clusters.** The free energy difference between the crystalline and amorphous phases is calculated as a function of the diameter of a spherical Au cluster in the crystalline phase.


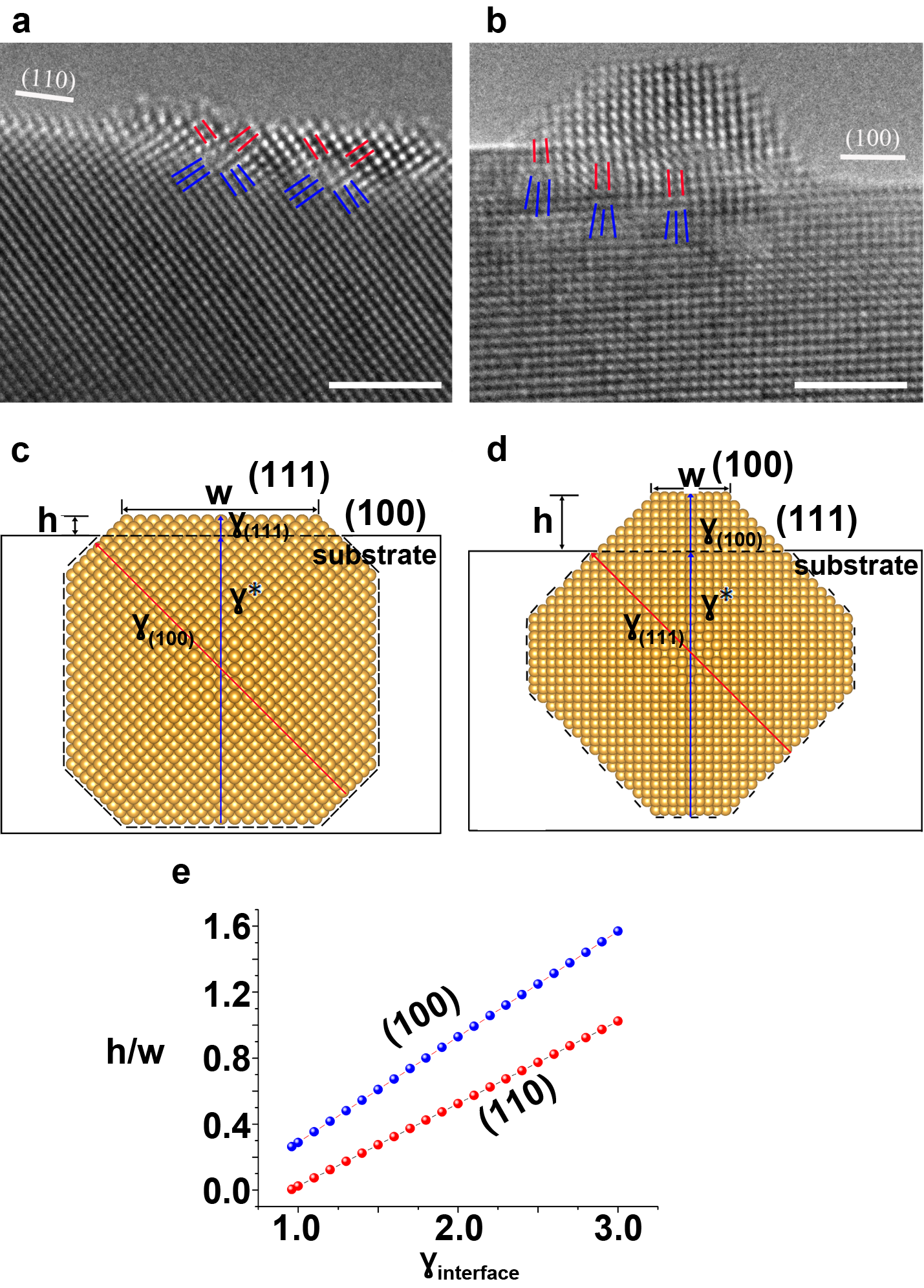


**Supplementary Figure 10: Equilibrium shapes of supported Au clusters**. **(a-b)** HRTEM images showing the equilibrium shapes of Au clusters on the (110) and (100) surfaces of the Cu(Au) solid solution, respectively. **(c-d)** Free Au clusters by the truncation parallel to the (111) and (100) surfaces. h, and w represent the height and width of the truncated cluster, respectively. $Ɣ^{*}$ represents the effective surface energy. **(e)** Plot of the aspect ratio as a function of the interface energy. Scale bar, 2 nm **a-b.**

**
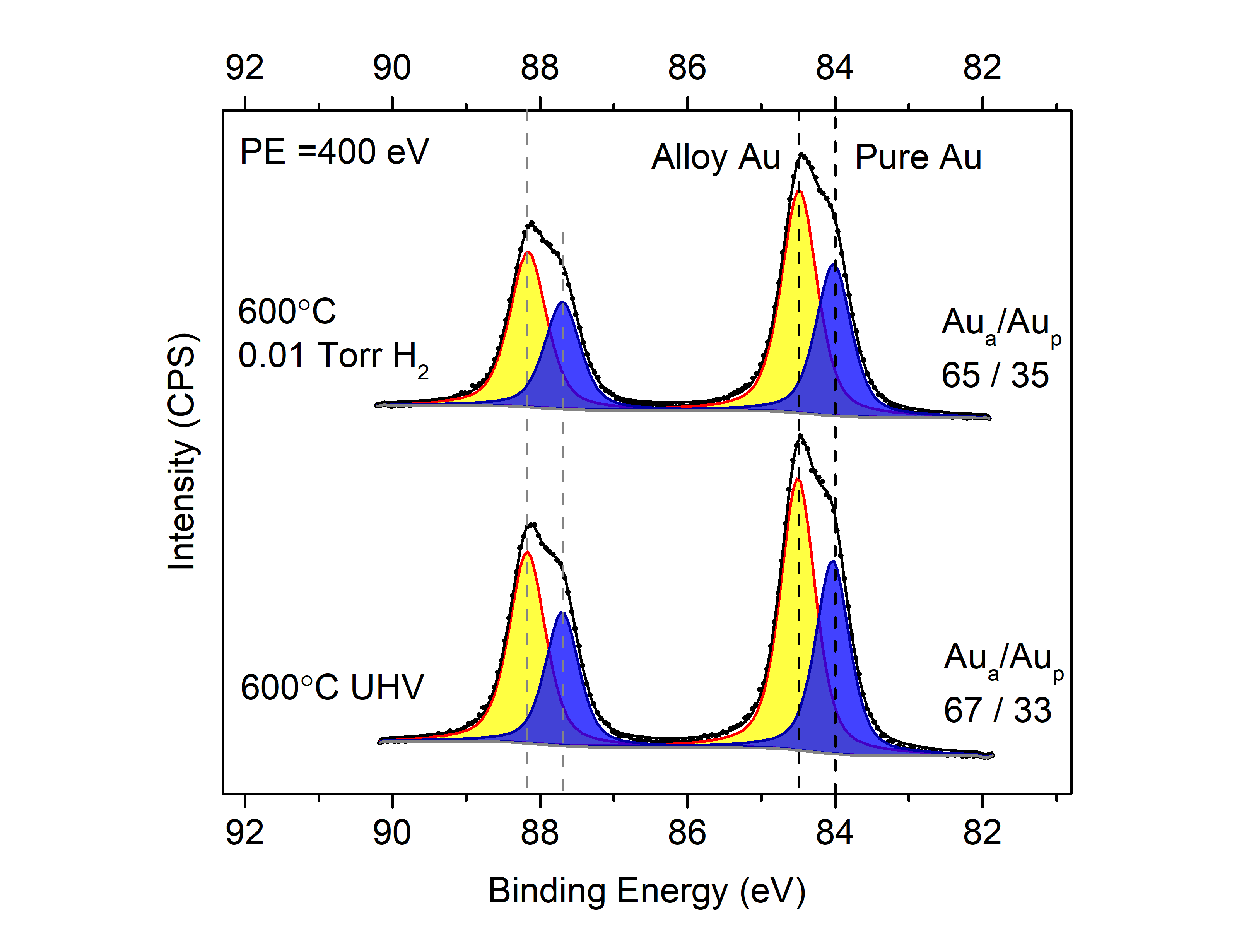
**

**Supplementary Figure 11:** **Comparison in surface Au composition of Cu_3_Au(100)**. The sample was annealing at 600 °C between 0.01 Torr of H_2_ gas flow (upper panel) and ultrahigh vacuum (UHV) (lower panel), respectively. Spectra were taken with the photon energy of 400 eV. The Au 4f region consists of two contributions corresponding to Au-4f_7/2_ and Au-4f_5/2_, respectively. Both contributions can be deconvoluted into two components, i.e., pure Au (Au_p_) and alloyed Au (Au_a_). The annealing under the H_2_ gas flow does not induce any notable differences in the pure Au concentration, as indicated by the Au_a_/Au_p_ ratio of integrated peak areas of each Au species.

**Supplementary Figure 12: STM images of the Cu_3_Au(100) annealed at ~ 600** °**C in ultrahigh vacuum. (a)** A typical surface area that is nearly free of clusters. **(b)** A separate surface area showing the presence of clusters. Scale bar, 20 nm **a, 10 nm b.**

**Supplementary Notes**

**Supplementary Note 1. Estimation of Au concentration in Au clusters by measuring the lattice spacing**

The relative composition of the Cu-Au alloys can be derived from the lattice spacing based on the Vegard’s law,

a=xa_Au_+(1-x)a_Cu_, (1)

where x represents the mole fraction of Au in the cluster, a_Au_(200)=2.04 Å, and a_Cu_(200)=1.807 Å. The measured d_(200)_ spacing of the cluster from the HRTEM image (Supplementary Figure 4) is a=2.01 Å. By inserting these values of the (200) lattice spacing into Eq. 1, we can find x=0.98. This indicates that the clusters are composed of nearly pure Au atoms.

**Supplementary Note 2. Surface steps as the active sources of Cu and Au adatoms**

Surface steps act as the active source of Au and Cu adatoms for the surface exchange processes as illustrated by *in situ* TEM observations (Supplementary Fig. 6). As seen from Supplementary Fig. 6(a), a surface step oriented along the <110> direction is present on the (001) surface. The surface step is observed to decay and undergo retraction from its original position by ~ 2.1 nm with a time period of 16 s, which results in a fluid phase of mobile Cu and Au adatoms detaching from the step edge. The disparity in the energy barriers for the exchange between these adatoms and the atoms in the substrate surface results in the extraction of Au atoms from the topmost layer of the substrate, thereby significantly enriching the concentration of Au adatoms in the fluid phase, as confirmed by the NEB simulations in Fig. 2 and KMC simulations in Supplementary Figure 7.

**Supplementary Note 3. Measurements of Au cluster rotation**

The in-plane rotation of the Au clusters can be measured either by tracking the orientation evolution of a specific set of lattice planes in real space or by monitoring the corresponding diffractograms in the reciprocal space. The latter provides the same quantitative information as the real-space lattice imaging. However, tracking in the real space can be more challenging if Au clusters undergo amorphous-crystalline oscillation. Here, we use the relative orientation of diffraction spots in the diffratograms to measure the rotation dynamics of clusters. Because the substrate maintains stationary without undergoing any rotation throughout the whole process, the substrate (220) spots are therefore selected as the reference. Supplementary Fig. 8 gives an example of the rotation dynamics as a function of time. The angle between two blue lines represents the in plane orientation between the Cu(Au) substrate and the Au cluster.

**Supplementary Note 4. Theoretical predication of the critical size of Au clusters for the amorphous to crystalline transition**

The critical size for the amorphous-crystalline transition in a Au cluster is evaluated by molecular dynamic simulations with the embedded-atomic-method potential[^1^](#_ENREF_2) as an energy calculator. Here, an amorphous Au matrix with a size of 10^3^ nm^3^ was constructed by heating crystalline Au to 2000 K and then quenched to 0 K. Then, a spherical nucleus of crystalline Au was used to replace the amorphous Au at the center of the matrix. The formation energy of the nucleus can be calculated by

$\Delta E_{f}=N_{nuc}(E_{nuc}-E_{matrix})$ (2)

where $E_{nuc}$ and $E_{matrix}$ are the energy per atom in the model of the matrix with and without the crystalline nucleus, respectively, and $N_{nuc}$ represents the number of atoms in the nucleus. The formation energy of eight cases with different nucleus diameters were investigated. For each diameter, the formation energy was averaged over 100 random configurations of the crystalline nucleus. The relation between the formation energy and nucleus diameter was fitted to the classical nucleation theory [^2^](#_ENREF_15) , e.g.,

$\Delta G(d)=\frac{\pi d^{3}\Delta G_{ac}}{6}+\pi d^{2}\sigma$ , (3)

where $\Delta G_{ac}$ is the free energy difference between the crystalline and amorphous states, and $\sigma$ is the interface energy. The critical size of crystalline Au is evaluated from the fitted $\Delta G_{ac}$ and $\sigma$ with the expression of

$d=\frac{4\sigma}{\Delta G_{ac}}$ . (4)

The corresponding plot is shown in Supplementary Fig. 9. In the plot, dE is defined as dE=(E_matrix+nucleation_-E_matrix_)/N_nucleation, where E_matrix+nucleation is the energy of the model with the crystalline nucleus, E_matrix is the energy of the matrix with the amorphous nucleus, N_nucleation is the number of atoms in the nucleus. From the fitted curve, the critical radius of the Au nucleus is predicted to be 2.6 nm in diameter, which is close to the diameter (~2.1nm) of the smallest crystalline Au clusters measured in the in-situ TEM experiments. Note that the method we used here did not incorporate the entropy effect. It is expected that the critical size can be underestimated because the entropy of amorphous phase is usually greater than that of the crystalline phase.

**Supplementary Note 5. Equilibrium shape of supported Au nanoclusters**

The equilibrium shape of a free Au cluster is constructed based on the Wulff construction using the surface energies of (100), (110), and (111) surfaces in reference [^3^](#_ENREF_16). The shape of the cluster, seen along the [100] direction (Supplementary Figs. 10(a, b)), is shown in Supplementary Figs. 10(c-d). For a supported Au cluster, the equilibrium shape can be described in a similar way by replacing the surface energy of a particular facet with the effective surface energy, Ɣ*, in which the effective surfae energy is defined as the energy difference between the interface and the support phase, Ɣ*= Ɣ_interface_-Ɣ_substrate_ ^4,5^. Based on the experimentally observed equilibrium shape, the support Au clusters located on the (110) and (100) surfaces of the substrate are truncated along the direction parallel to the (111) facet, and (100) facet, respectively, as shown in Supplementary Fig.. 10(c and d). A straightforward analysis on the geometry gives the expression of the aspect ratio of the supported cluster, h/w, as a function of the effective energy (Eqs. 8 and 9).

For the (110) supported Au cluster:

$\frac{h}{w}$ = $\frac{Ɣ_{1}^{*}+Ɣ_{111}}{Ɣ_{100}}$ $\sqrt{\frac{2}{3}}$ (5)

For the (100) supported Au cluster:

$\frac{h}{w}$ = $\frac{Ɣ_{2}^{*}+Ɣ_{100}}{Ɣ_{111}}$ $\sqrt{\frac{2}{3}}$ (6)

$Ɣ_{1}^{*}$ and $Ɣ_{2}^{*}$ represent the effective surface energy of the (111)/(110) and (100)/(100) system, respectively. By putting the surface energies of the Au cluster with the values of $Ɣ_{111}$=1.283 J/m^-2^, and $Ɣ_{100}$=1.627 J/m^-2^ ^3^ into Eqs. (5) and (6), the above two equations can be converted to:

For the (110) supported Au cluster:

$\frac{h}{w}$ = $0.5Ɣ_{1}^{*}+0.643$ (7)

For the (100) supported Au cluster:

$\frac{h}{w}$ = $0.64Ɣ_{2}^{*}+1.035$ (8)

Considering Ɣ*= Ɣ_interface_-Ɣ_substrate_, in which Ɣ_substrate_ is a constant, with Ɣ_substrate_ = 2.237J/m^-2^ for the (111)/(110) alignment, and Ɣ_substrate_ = 2.166 J/m^-2^ for the (100)/(100) alignment, the $\frac{h}{w}$ is solely determined by the interface energy Ɣ_interface_.

For the (110) supported Au cluster:

$\frac{h}{w}$ = 0.5 Ɣ_interface_ -0.476 (9)

For the (100) supported Au cluster:

$\frac{h}{w}$ = $1$0.64 Ɣ_interface_ -0.351 (10)

When $\frac{h}{w}$ decreases, the cluster gradually transists to a 2D shape, otherwise, it transits to a 3D shape. Therefore, the wetting behavior of the supported Au cluster can be described by parameter Ɣ_interface_. The aspect ratio as function of the effective energy is plotted within the range of [0.95 2]. As can be seen from Supplementary Fig. 10e, there are two factors that contribute to the 2D-like shape of the (110)-supported Au cluster while the 3D-like shape of the (100)-supported cluster:

(1) surface energies. Because of the higher surface energy of the (100) surface than that of the (111) surface for Au, which is also applied for most of FCC metals, the aspect ratio of the (100)-supported Au cluster is always higher than the (110)-supported cluster in the case that the interface energy is the same, indicating the intrinsic tendency to form a more 3D-like shape for the support cluster on the (100) surface.

(2) Interface dislocation properties. The interface energies can be largely affected by the interfacial dislocations. As can be seen from our in-situ TEM observations, the Au cluster on the (100) surface tends to form misfit dislocations with the [100]-type Burgers vector while the Au cluster on the (110) surface results in misfit dislocations with the [110]-type Burgers vector. Often, the interfacial energy relief scales with the magnitude of the Burgers vector. The (110)-supported Au cluster releases more interfacial energy than that of the (100)-supported Au clusters because of the larger Burgers vector for the misfit dislocations at the (110) interface. Therefore, the Au clusters on the (110) surface develop into a more 2D-like wetting layer than the cluster on the (100) surface.

**Supplementary Note 6. Excluding possible electron-beam effects on in-situ TEM observations**

The procedures for estimating the electron beam effects on TEM observations were well-established by Egerton, et. al [^6^](#_ENREF_19). We have followed the established procedures to quantify the possible beam effects on the Cu-Au system, and the analysis leads to the same conclusion as the previous work [^7^](#_ENREF_20), i.e., the electron beam has negligible effects on the observed phenomenon. According to Egerton et al., possible electron beam effects on TEM observations include charging, heating, atom displacement, sputtering, and radiolysis, below we analyze the effects on the Cu-Au systems one by one.

(i) Charging: Charging effect is expected to be trivial due to the high conductivity of both Cu and Au.

(ii) Heating: One of the major effects that contribute to the heating of the system is the inelastic collision of the beam electrons and Cu/Au electrons, which is estimated by [^6^](#_ENREF_19),

$\Delta T=I\cdot\left\langle E(eV) \right\rangle4\pi\kappa\lambda\left[ 0.58+2ln (2R0/d) \right]$ , (11)

in which I is the electron beam current, 〈E(eV)〉 is the average energy loss per inelastic collision, λ is the mean free path for in elastic scattering, κ is the thermal conductivity of the material, d is the incident beam diameter, R0 is the heat conduction distance. In our experiments, the beam current is ~2.1 nA, 〈E(eV)〉 is 50~100 eV (plasma peak of the electron energy loss spectrum), ~314 W/m/K (for Au), ~385 W/m/K (for Cu substrate), λ is ~84 nm (for Au), ~100 nm (for Cu substrate) , beam diameter d is ~600 nm, heat dissipation radius R0 is ~3 mm. Putting the values of these parameters into the equation gives a temperature rise of <1 K, which is negligible relative to the annealing temperature (600 °C).

(iii) Atomic displacement: The atomic displacement is typically considered to be related to elastic knock-on of the electron beam, generating vacancies and interstitial atoms. The maximum energy that can be adsorbed by the system can be estimated by[^6^](#_ENREF_19),

$E_{max}= \frac{E_{0} (1.02+\frac{E_{0}}{{10}^{6}})}{465.7 A}$ (12)

where E_0_ is the incident energy of electron in eV, A is the mass number of the element. In our experiments, E_0_ is ~300 keV, A is 197 for Au. This gives a maximum transfer energy of ~4.3 eV which is well below the threshold displacement energy of Au ~34 eV [^6^](#_ENREF_19). Therefore, displacement effect is also trivial in this system.

(v) Sputtering: The sputtering can be triggered when the E_max_ is larger than the threshold value of sample (E_s_) which can be estimated from sublimation energy. Considering E_max_ (~4.3 eV) > E_s_ of Au (~3.8 eV/atom), the sputtering is a possible event in our experiments, the rate of which can be estimated by[^3^](#_ENREF_3),

$S =\frac{J}{e} \frac{Z^{2}}{AE_{0}}(\frac{1}{E_{s}}-\frac{1}{E_{max}})(3.54\times{10}^{-17}\mathrm{cm}^{2})$ (13)

where J/e is the electron dose rate, Z is the atomic number (79 for Au). Putting the dose rate of ~3×10^19^ electrons/cm^2^/s (~4 A/cm^2^) into the equation gives a sputtering rate of ~0.0032 monolayer/s. Given the typical TEM image acquisition rate (2 frames per second) in our experiments, the sputtering effects on the TEM observations are negligible.

**Supplementary Note 7. Excluding effect of hydrogen gas flow on the surface composition**

Ambient-pressure photoelectron spectroscopy (AP-XPS) was performed on Cu_3_Au(100) annealed at 600 °C under ultrahigh vacuum (UHV) and in 0.1 Torr of H_2_ gas flow (Supplementary Fig. 11). The AP-XPS experiments were performed at the IOS beamline of the National Synchrotron Light Source II (NSLS-II), Brookhaven National Laboratory. More experimental detail can be found from our previous work ^8, 9, 10^. XPS spectra were analyzed using a Shirley-type background with the Voigt function. Line shape of a Gaussian/Lorentzian sum formula modified by the exponential blend was introduced for accurate peak fitting and deconvolution. Full width at half-maximum (FWHM) of Au is 0.5-0.6 eV for alloyed Au and 0.4-0.5 eV for metallic Au. Integrated peak areas of each Au species were used to calculate the relative composition evolution. The Cu_3_Au(100) single crystal (Princeton Scientific Corp, purity = 99.9999%) is a top-hat-shaped disc (1 mm thick and 8 mm in diameter), cut to within 0.1° to the (100) crystallographic orientation and polished to a mirror finish. The crystal was heated via a ceramic button heater and its temperature was monitored with a type-K thermocouple. The pristine Cu_3_Au(100) crystal was cleaned by repeated cycles of Ar-ion^+^ bombardment at room temperature followed by UHV annealing (550 °C, 10 min) until no O 1s and C 1s spectra could be detected using XPS. A separate system equipped with the capabilities for surface structure determination, that is, low-energy electron diffraction and scanning tunneling microscopy, was used to check the surface quality of the Cu_3_Au(100) prepared using the same sputtering and annealing procedure as the AP-XPS experiments. High-purity hydrogen gas (purity = 99.9999%) was directly introduced to the sample compartment through a variable-pressure leak valve.

**Supplementary Note 8. Comparison between Cu-10at.%Au(100) and intermetallic Cu_3_Au(100)**

The same stoichiometric, intermetallic Cu_3_Au(100) single crystal used in the AP-XPS experiments (see Supplementary Fig. 11) was also examined by scanning tunneling microscopy (STM) by the UHV annealing of the crystal at ~ 600 °C. The crystal was heated via a ceramic button heater and its temperature was monitored with a type-K thermocouple. The Cu_3_Au(100) crystal was cleaned by repeated cycles of Ar-ion^+^ bombardment at room temperature followed by UHV annealing (~600 °C, 10 min). The surface structure and morphology were monitored by low-energy electron diffraction and STM imaging at room temperature (Supplementary Fig. 12).

**Supplementary References:**

1 Foiles S, Baskes M, Daw MS. Embedded-atom-method functions for the fcc metals Cu, Ag, Au, Ni, Pd, Pt, and their alloys. *Phys. Rev. B* **33**, 7983 (1986).

2 Abraham F. Homogeneous Nucleation Theory,(1974).). Academic Press, New York (2008).

3 Vitos L, Ruban A, Skriver HL, Kollar J. The surface energy of metals. *Surf. Sci.* **411**, 186-202 (1998).

4 Hansen KH*, et al.* Palladium nanocrystals on Al 2 O 3: structure and adhesion energy. *Phys. Rev. Lett.* **83**, 4120 (1999).

5 Winterbottom W. Equilibrium shape of a small particle in contact with a foreign substrate. *Acta Metall.* **15**, 303-310 (1967).

6 Egerton R, Li P, Malac M. Radiation damage in the TEM and SEM. *Micron* **35**, 399-409 (2004).

7 He Y*, et al.* Size-dependent dynamic structures of supported gold nanoparticles in CO oxidation reaction condition. *Proc. Natl. Acad. Sci.* **115**, 7700-7705 (2018).

8 Li C, Liu Q, Boscoboinik JA, Zhou G. Tuning the surface composition of Cu_3_Au binary alloy. *Phys. Chem. Chem. Phys.* **22**, 3379-3389 (2020).

9 Li C, Zhang P, Wang J, Boscoboinik JA, Zhou G. Tuning the deoxygenation of bulk-dissolved oxygen in copper. *J. Phys. Chem. C* **122**, 8254-8261 (2018).

10 Wang J, Li C, Zhu Y, Boscoboinik JA, Zhou G. Insight into the phase transformation pathways of copper oxidation: from oxygen chemisorption on the clean surface to multilayer bulk oxide growth. *J. Phys. Chem. C* **122**, 26519-26527 (2018).
