# Supplementary material for: Atomic-scale phase separation induced clustering of solute atoms
Source: Nat Commun. 2020 Aug 7;11:3934. doi: 10.1038/s41467-020-17826-w (PMC7415157; doi:10.1038/s41467-020-17826-w)
Supplement: Supplementary file 3 — Description of Additional Supplementary Files [file 41467_2020_17826_MOESM3_ESM.pdf]

Description of Additional Supplementary Files

File Name: Supplementary Movie 1
Description: Animation showing the atomic processes leading to the clustering of Au adatoms, i) the formation of a fluid phase of Cu and Au adatoms by step-edge detachments, ii) exchanges between adatoms in the fluid phase and substrate atoms, resulting in the enrichment of Au adatoms in the fluid phase, iii) clustering of Au adatoms in the fluid phase

File Name: Supplementary Movie 2
Description: In-situ TEM video showing the nucleation, growth, crystallization and rotation of Au clusters

File Name: Supplementary Movie 3
Description: In-situ TEM video showing the crystalline-to-amorphous transition in an Au cluster

File Name: Supplementary Movie 4
Description: In-situ TEM video showing the crystalline-to-amorphous transition in another Au cluster

File Name: Supplementary Movie 5
Description: In-situ TEM video showing the amorphous-to-crystalline transition in an Au cluster

File Name: Supplementary Movie 6
Description: Molecular dynamics (MD) simulations showing the atomic structure evolution in an Au cluster that is misaligned with the substrate by 15° between the (110) lattice planes of the cluster and substrate

File Name: Supplementary Movie 7
Description: MD simulations showing the atomic structure evolution in an Au cluster that is misaligned with the substrate by 90° between the (110) lattice planes of the cluster and substrate

File Name: Supplementary Movie 8
Description: MD simulations showing the atomic structure evolution in an Au cluster that is perfectly aligned (0°) with the substrate between the (110) lattice planes of the cluster and substrate
